# Risk Factors Associated with Passenger Vehicle Fatal Rollover Crashes in West Virginia, 2001–2018

**DOI:** 10.13023/jah.0304.05

**Published:** 2021-10-25

**Authors:** Yuni Tang, Toni Marie Rudisill, Ruchi Bhandari

**Affiliations:** Department of Epidemiology, School of Public Health, West Virginia University, Morgantown WV

**Keywords:** Appalachia, motor vehicle crashes, rollover crashes, public health, Fatality Analysis Reporting System (FARS), multivariable logistic regression

## Abstract

**Background:**

Rollover crashes cause more injuries and fatalities than other types of motor vehicle crashes. West Virginia (WV) has high rates of drug overdose deaths and motor vehicle crash fatality. However, no studies have investigated risk factors associated with fatal rollover crashes in WV.

**Purpose:**

The objective of this study is to evaluate whether drug use and other risk factors are associated with fatal rollover crash fatalities in WV.

**Methods:**

This cross-sectional study utilized the Fatality Analysis Reporting System dataset from passenger vehicle crashes involving WV drivers ≥ 16 years of age with known drug test results who died within 2 hours after collision from 2001 to 2018. Risk factors associated with fatal rollover crashes were compared to non-rollover crashes using multivariable logistic regression.

**Results:**

During the study period, 880 WV drivers died in rollover crashes. Driving ≥ 60 mph [adjusted odds ratio (aOR): 4.1; 95% confidence interval (CI): 2.4–6.8], alcohol use (aOR: 1.6; 95% CI: 1.1–2.1), rural areas (aOR: 1.4; 95% CI: 1.0–1.9), and the lack of airbag deployment (aOR: 2.7; 95% CI: 2.1–3.5) were associated with fatal rollover crashes in WV. However, drug use was not associated with fatal rollover crashes in the final multivariable logistic regression model (aOR:1.13; 95% CI: 0.9–1.5).

**Implications:**

Findings of risk factors associated with rollover crash fatalities in WV can inform several public health interventions. Rapid and sensitive assessment tools and standardized toxicology testing are helpful to provide more comprehensive drug-impaired driving datasets for future analysis.

## INTRODUCTION

Motor vehicle crashes are a major concern globally, as well as in the U.S., due to the irreversible impacts on human loss and property damage. In 2018, 36,560 people in the U.S. were killed in motor vehicle crashes.^1^ One of the most severe motor vehicle crash events is a rollover; rollover crashes cause more injuries and fatalities than other crash types, even though they account for a low proportion of all crashes.^2^,^3^ A total of 6358 passenger vehicle crashes were involved in a rollover during 2019, but rollovers accounted for nearly 28% of all passenger vehicle crash deaths.^4^ The number of deaths due to motor vehicle crashes in West Virginia (WV) during 2019 was 260, which is an 11.6% decrease from 294 deaths in 2018.^5^

Rollovers occur when a vehicle rotates at least one-quarter turn that is greater than or equal to 90 degrees about the vehicle’s lateral or longitudinal axis with the majority occurring about the longitudinal axis.^3^ Those who failed to use restraints, were younger drivers, and drove at higher speeds had a higher likelihood of experiencing fatal rollover crashes.^6^,^7^ Additionally, rollover crashes were more likely to occur on rural, curved roads compared to urban, straight roads.

A study conducted in Ohio found that drug or alcohol use was also a risk factor for rollover crashes.^8^ In 2018, the number of people who died in alcohol-impaired driving crashes accounted for nearly 29% of total traffic fatalities.^1^ While driving under the influence of alcohol is a well-documented traffic safety threat, drugged driving is also becoming a major public health issue in the U.S. and has increased between 1993 and 2010.^9^ Approximately 28% of fatally injured U.S. drivers tested positive for one or more of illicit or legal drugs in 2009.^10^ The prevalence of U.S. drivers with non-alcohol drug use in fatal vehicle crashes has increased significantly with marijuana and prescription drugs, particularly with opioids becoming more prevalent.^9^–^12^ These substances are known to affect driver performance by affecting reaction times, causing drivers to commit signal violations, and decrease cognitive functioning.^13^

Compared to other states, WV has the highest age-adjusted rate of drug overdose deaths and a higher rate of motor vehicle fatalities.^4^,^14^ The purpose of this study was to determine whether there is an association between drug use and rollover crashes in WV from 2001 to 2018. In addition, the second aim of this study was to evaluate other risk factors associated with fatal rollover crashes in the state. The results of this study could inform future interventions to decrease or prevent future rollover crash injuries and fatalities in WV. As this study focused on decedent drivers, it did not qualify as human subjects’ research by the Institutional Review Board at West Virginia University.

## METHODS

The data used for this cross-sectional study were obtained from the Fatality Analysis Reporting System (FARS) for calendar years 2001 to 2018. The FARS is a cross-sectional database of all fatal traffic crashes which occurred in the U.S.^15^ The FARS contains information on persons, vehicles, crash characteristics, and events. To qualify as a FARS case, the crash must involve a motor vehicle on a public traffic way where at least one person dies in the crash within 30 days of the incident.^15^ Prior to 2018, up to three tests and associated types of drugs for each individual involved in the traffic collisions were documented in FARS, in addition to a blood alcohol concentration; nicotine, aspirin, alcohol, and drugs administered after the collision are excluded from FARS cases.^10^ The FARS began reporting all drugs detected in a driver starting in 2018.^15^ Drug tests were administrated by obtaining blood and/or urine samples. The FARS data do not include any personal identifiers and are publicly available at http://www.nhtsa.gov/FARS.

**Figure 1:**
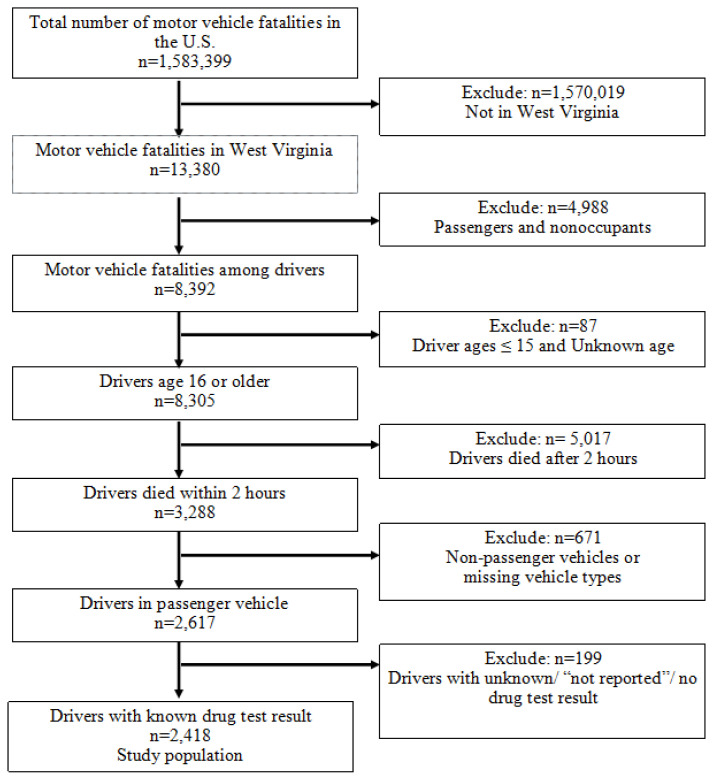
Flow chart of study population based on the Fatality Analysis Reporting System (FARS), 2001–2018

The study population was limited to drivers ≥16 years of age who died in a passenger vehicle crash that occurred in WV and had a known drug test result. According to the vehicle type definition in FARS, passenger vehicles included passenger cars, pickups, utility vehicles, and vans. Therefore, drivers of large and medium trucks, motorcycles, buses, farm equipment, large limousines, all-terrain vehicles, and other unknown type vehicles were excluded from the analysis.^15^ The study population was further limited to drivers who died within 2 hours of collision. This was done to minimize the possibility of drugs administered post-collision being reported in the drug test results. Drivers with no drug test result or with missing and unknown drug test results were also excluded. The flow chart of the study population showed in Figure 1.

The dependent variable, rollover crash, was defined as any vehicle rotation of 90 degrees or more in the longitudinal or lateral axis, both tripped and untripped by object or vehicle.^15^ This variable was dichotomized as: (1) rollover crash; and (2) nonrollover crash. Independent variables known to be associated with rollover motor vehicle crashes were chosen based on previous studies and availability in the FARS dataset.^8^,^16^,^17^ In this study, all independent variables were categorized as driver, environmental, and/or vehicle characteristics. The classification and coding in FARS of each variable used in the analyses are in Table 1.

Descriptive analyses consisted of fatality counts as well as percentage for all the exposure variables stratified by rollover crash vs. nonrollover crash. All binary variables were analysed using Chi-square tests. Cochran-Armitage Trend tests with Modified Ridit scoring were used to analyse all ordinal variables. The multivariable logistic regression model is commonly used in epidemiological studies, including those investigating motor vehicle collisions, when the outcome is binary.^18^ In this study, two multivariable logistic regression models were analysed to calculate adjusted odds ratios (aOR) and 95% CIs for each covariate and the outcome variable. Multivariable logistic regression model selection was based on descriptive analysis with p-value ≤0.20, which is commonly used in epidemiologic research for variable inclusion criteria in the model. Model 1 examined the association of driver characteristics (age group, gender, blood alcohol concentration [BAC], restraint use, and known drug test result) with the outcome variable. Model 2 adjusted for all eligible exposure variables based on bivariate analysis. The effective sample size of Model 1 was N=2173 and Model 2 was N=1634 after excluding participants with missing data. In addition, a sensitivity analysis was conducted *post hoc* to examine the association between vehicle age and airbag deployment due to a large number of missing values of airbag deployments. The aim of the sensitivity analysis was to determine if the vehicles involved int these crashes were old and may not have had airbags. The vehicle age was defined as “Year of crash – Model year” (shown in Table 1). Another sensitivity analysis was conducted to estimate the association between covariates and fatal rollover crash status adding drivers who died after 2 hours of collision during the study period (Supplement Table 1; see Additional Files). All analyses were conducted using SAS, version 9.4. Two-tailed hypothesis tests were utilized with α=0.05.

## RESULTS

Overall, 2418 drivers met the inclusion criteria (Figure 1). Descriptive characteristics of rollover motor vehicle crash fatalities are summarized in Table 2. Of them, 880 (36.4%) drivers died in rollover crashes and 1538 (63.6%) died in nonrollover crashes. Among the drivers who died in rollover crashes, the majority were male, not belted, and did not have previous DWI convictions (94.8%), previous speed convictions (83.4%), or previous recorded crashes (90.6%). Two-thirds of fatal rollover crashes occurred when the speed limit was between 35 mph and 55 mph (66.5%); most crashes occurred in rural areas (82.1%). Over 90% of fatal rollover crashes occurred on blacktop, bituminous, or asphalt surface type, and 18% occurred during adverse weather conditions. Passenger cars (n=353, 40%) and single-vehicle crashes (n=770, 87.5%) accounted for the majority of fatal rollover crashes in WV.

The aORs and 95% CIs were calculated in two different multivariable logistic regression models, which are shown in Table 3. Due to drivers with missing data on exposure variables, 245 participants in Model 1 and 784 participants in Model 2 were excluded. After controlling for all the driver characteristic variables in Model 1, drivers with BAC ≥ 0.08 g/dl (aOR:2.14; 95% CI:1.74–2.61) and without restraints (aOR:1.70; 95% CI:1.39–2.08) had higher odds of fatal rollover crashes in comparison with other nonrollover crashes. In Model 2, after adjusting for all covariates, there was a significant association between fatal rollover crashes and BAC ≥ 0.08 g/dl (aOR:1.56; 95% CI:1.14–2.14), as well as driving on a road with a 60 mph speed limit (aOR:4.06; 95% CI:2.42–6.78), crashing in a rural area (aOR:1.39; 95% CI:1.02–1.89), driving a utility vehicle (aOR:2.44; 95% CI:1.76–3.39), pickups (aOR:1.54; 95% CI:1.12–2.12), being involved in a single-vehicle collision (aOR:8.37; 95% CI:6.18–11.34), and no airbag deployment (aOR:2.71; 95% CI:2.10–3.51).

In the first sensitivity analysis (not shown), vehicles over 10 years of age were more likely to lack air bag deployment information than newer vehicles (odds ratio [OR]:1.70; 95% CI:1.39–2.08). However, there were no significant associations between vehicle age and fatal rollover crashes (OR: 1.02; 95% CI: 0.86–1.21). Another sensitivity analysis investigated the association between eligible covariates and fatal rollover collisions among all drivers who died in collisions. The positive drug test results had a significant association with fatal rollover crashes (aOR:1.24; 95% CI: 1.05, 1.46) in Model 1. After adjusting for all covariates, there was a significant association between fatal rollover crashes and drivers who did not use seat belt (aOR:1.29; 95% CI: 1.02, 1.64), as well as driving on a road at 35–55 mph speed limit (aOR:1.64; 95% CI: 1.10, 2.45) (Supplement Table 1; see Additional Files).

## IMPLICATIONS

This study sought to determine driver, vehicular, and environmental risk factors associated with fatal rollover crashes in WV using 18 years of FARS data. While characteristics of rollover crashes have been documented in other studies, they have not been documented for fatal rollover crashes specifically in West Virginia.^7^,^8^,^16^–^18^ This information is important to garner from a public health perspective as crash fatality rates are higher in this state.^19^ This study determined that most fatal rollover crashes in WV were associated with drivers who were travelling on a high-speed roadway, crashed in a rural area, involved in single-vehicle collisions, operating a utility or pickup truck, and experienced no air-bag deployment, compared with nonrollover collisions. Drivers involved in fatal rollover collisions were found to be positive for alcohol but not for drugs.

The findings in this study were partially consistent with results from other studies; other studies have also found that alcohol consumption, driving speed, operating a truck/utility vehicle, and lacking airbags are risk factors for rollover crashes.^7^,^8^,^16^,^17^ Alcohol is known to impair drivers’ cognition and driving performance making them more prone to collision. Higher speeds and vehicles with high centers of gravity, such as trucks and utility vehicles, may also make vehicles more prone to tip. As a rural, mountainous state, WV’s roads are often windy, narrow in width, and may be undivided/lack barriers. Drivers could lose control of their vehicles at high speeds in these locations.^16^ Airbags have played an important role in protecting drivers and occupants and can improve vehicles’ crash worthiness.^8^,^17^ Due to the special topography in WV, more fatal rollover crashes occurred in rural areas compared to urban areas; rurality was identified as a risk factor for fatal rollover crashes in other studies as well.^8^,^16^,^17^,^20^ Also, longer emergency medical services (EMS) response time and limited EMS locations in rural areas may explain the high likelihood of deaths following a motor vehicle crash.^21^,^22^ Although night-time was not statistically significant in this study, the rollover fatalities tend to occur more often at night time due to potential fatigue-driving and less traffic volume at night time.^17^ Additionally, the poor lighting conditions at night may affect driver’s vision that result in severe rollover crashes, especially in rural areas without roadside lights. There was a contradiction to the association between the day of week and fatal rollover crashes.^6^, ^17^ Fatal rollover crashes occurred more on weekends due to people experience more alcohol-involved driving compared to weekdays.^6^ However, another study showed that fatal rollover crashes occurred more on weekdays due to higher traffic volume during the work week.^17^

However, this study has some limitations. First, the temporal or causal relationship between risk factors and rollover crashes cannot be established due to the cross-sectional nature of the data. Second, there are well-known limitations of FARS data especially involving drug use. For example, although a positive drug test result indicates that the driver consumed a specific type of drug, it cannot conclude that the driver was impaired by the drug at the time of the rollover crash.^10^ Also, our definition of drug use was very broad. Third, there are at least two sources of potential selection bias. This study selected drivers who died in rollover crashes with known drug test results which could result in a selection bias. However, potential bias would be minimized because WV tests a large proportion of fatally injured drivers for alcohol and/or drugs.^23^ In addition, although FARS does not include drivers who use/receive drugs after the collision, due to the complicated nature of injury severity and mechanism, there is a possibility that some of these drivers may still be included in the FARS data. Thus, in order to minimize this specific selection bias, we limited drivers who died within 2 hours after rollover collision, which is a realistic timeframe that first responders may need to reach the victim in a rural state. Fourth, although airbag deployment was associated with rollover collisions in WV, 24% of fatal rollover crashes did not have airbag deployment information because not all vehicles have side or curtain airbag deployment algorithms for rollover crashes, particularly older vehicles do not have the airbag sensor. This could be due to the extensiveness of vehicle damage which occurs in rollover events. Also, the possibility of overfitting the model exists when adding more exposure variables in the multivariable logistic regression model, although it reduces the possibility of residual confounding. Moreover, this study only included fatal rollover crashes in West Virginia; the risk factors identified in this study may not apply to nonfatal rollover crashes. The final limitation is the generalizability of the results as this study was limited to WV. The study results cannot represent the fatal rollovers in other states without the same geographic characteristics.

Even though rollover crashes often result in severe injuries and/or death, many risk factors associated with these fatal rollover collisions in this study are modifiable and several opportunities exist for intervention in West Virginia. Measures such as reducing speed limits, posting appropriate speed limits and roadside warning signs, and driver education have shown to be helpful in preventing rollover collisions.^3^,^8^,^17^ Lowering the BAC limit from 0.08 g/dl to 0.05 g/dl and conducting publicized sobriety checkpoints regularly can be effective strategies to reduce or prevent alcohol-impaired driving. To increase seat belt use in West Virginia, public health campaigns are needed. Additionally, penalties for driving without using seat belt can be applied and beneficial to increase seat belt usage^8^. Roadside warning signs for fatigued driving at night and improved roadway lighting on rural roads could be beneficial. This present study did not find significant association between drug use and fatal rollover crashes in West Virginia, but rapid and sensitive drug assessment tools and standardized toxicology testing are helpful to provide more comprehensive drug data for future drug-impaired driving studies.

**Summary Box**

**What is already known about this topic?** Compared to other types of motor vehicle collisions, rollover crashes account for a considerable number of fatalities. Some known risk factors, such as alcohol consumption, single-vehicle collisions, and driving a utility or pickup truck, are associated with fatal rollover collisions.

**What is added by this report?** This is the first study to investigate risk factors, including drug use, associated with fatal rollover crashes in West Virginia. Drug use was not a significant risk factor for rollover crashes in comparison with other crashes. Rurality and high speeds were identified as main risk factors for fatal rollover crashes due to the specific geographic characteristics in West Virginia.

**What are the implications for public health practice, policy, and research?** Findings highlight that many risk factors associated with fatal rollover collisions are modifiable and several opportunities exist for preventing rollover collisions in West Virginia. Rapid and sensitive drug assessment tools and standardized toxicology testing are needed to provide more comprehensive data for future drug-impaired driving studies.

## Supplementary Information



## Figures and Tables

**Table 1:** List of variables included in statistical analyses

Variables	Description	FARS codes
**Dependent variable**	Rollover crashes	ROLLOVER
	Nonrollover crashes	
**Independent variables**		
Driver characteristics	Age	AGE
	Gender	SEX
	BAC*	ALC_RES
	Restraint use	REST_USE
	Drug test result	DRUGES1, DRUGRES2, DRUGES3(2001–2017) DUGRES (2018)
	DWI* convictions	PREV_DWI
	Speeding convictions	PREV_SPD
	Previous recorded convictions	PREV_ACC
Environmental characteristics	Speed limit	SP_LIMIT (2001–2009) VSPD_LIM (2010–2018)
	Land use	ROAD_FNC (2001–2014) RUR_URB (2015–2018)
	Day of week	DAY_WEEK and HOUR
	Time of day	HOUR
	Year group	YEAR
	Roadway alignment	ALIGNMNT (2001–2009) VALIGN (2010–2018)
	Roadway grade	PROFILE (2001–2009) VPROFILE (2010–2018)
	Roadway surface type	PAVE_TYP (2001–2009) VPAVETYP (2010–2018)
	Adverse weather	WEATHER
Vehicle characteristics	Vehicle type	BODY_TYP
	Number of vehicle crash	VE_FORMS
	Airbag deployed†	AIR_BAG
	Vehicle age§	“YEAR - MOD_YEAR ”

* Abbreviations: Blood Alcohol Concentration; Driving While Impaired.

† Defined as airbag deployed from front (steering wheel, dashboard), side (door, seat, canopy), other direction (knee, air belt), multiple directions, and unknown direction.

§ Vehicle age is used for sensitivity analysis and defined as “Year of crash – Model year”

**Table 2:** Characteristics of Fatal Rollover Crashes in WV, 2001–2018*

Variables	Rollover 880 (36.4%)	Nonrollover 1538 (63.6%)	Total 2418 (100%)	p-value†
**Driver characteristics**				
**Age**				**0.0009**
16–24	206 (23.4%)	311 (20.2%)	517 (21.4%)	
25–44	341 (38.8%)	528 (34.3%)	869 (35.9%)	
≥45	333 (37.8%)	699 (45.5%)	1032 (42.7%)	
**Gender**				**0.0001**
Male	666 (75.7%)	1052 (68.4%)	1718 (71.1%)	
Female	214 (24.3%)	486 (31.6%)	700 (29.0%)	
**Blood Alcohol Concentration**				**<0.0001**
0.00	472 (53.6%)	1135 (73.8%)	1607 (66.5%)	
0.01–0.07	32 (3.6%)	48 (3.1%)	80 (3.3%)	
≥0.08	374 (42.5%)	351 (22.8%)	725 (30.0%)	
Missing	2	4	6	
**Restraint Use**				**<0.0001**
Belted	202 (23.0%)	571 (37.1%)	773 (32.0%)	
Not belted	611 (69.4%)	794 (51.6%)	1405 (58.1%)	
Unknown	67	173	240	
**Drug Test Result**				**0.0238**
Positive	336 (38.2%)	517 (33.6%)	853 (35.3%)	
Negative	544 (61.8%)	1021 (66.4%)	1565 (64.7%)	
**DWI**§ **convictions**				**0.5529**
Yes	37 (4.2%)	57 (3.7%)	94 (3.9%)	
No	834 (94.8%)	1460 (94.9%)	2294 (94.9%)	
Missing	9	21	30	
**Speeding convictions**				**0.8436**
Yes	137 (15.6%)	234 (15.2%)	371 (15.3%)	
No	734 (83.4%)	1283 (83.4%)	2017 (83.4%)	
Missing	9	21	30	
**Previous recorded crashes**				**0.5203**
Yes	73 (8.3%)	139 (9.0%)	212 (8.8%)	
No	797 (90.6%)	1377 (89.5%)	2174 (89.9%)	
Missing	10	22	32	
**Environmental characteristics**				
**Speed Limit**				**0.0002**
30 or less	58 (6.6%)	110 (7.2%)	168 (7.0%)	
35 to 55	566 (64.3%)	1106 (71.9%)	1672 (69.2%)	
60 or more	227 (25.8%)	286 (18.6%)	513 (21.2%)	
Missing¶	29	36	65	
**Land use**				**0.0009**
Rural	722 (82.1%)	1178 (76.6%)	1900 (78.6%)	
Urban	155 (17.6%)	360 (23.4%)	515 (21.3%)	
Missing	3	0	3	
**Day of week**				**<0.0001**
Weekdays	517 (58.8%)	1039 (67.6%)	1556 (64.4%)	
Weekends	363 (41.3%)	499 (32.4%)	862 (35.7%)	
**Time of day**				**<0.0001**
Daytime	389 (44.2%)	917 (59.6%)	1306 (54.0%)	
Nighttime	491 (55.8%)	621 (40.4%)	1112 (46.0%)	
**Year group**				**0.9305**
2001–2006	324 (36.8%)	579 (37.7%)	903 (37.3%)	
2007–2012	313 (35.6%)	517 (33.6%)	830 (34.3%)	
2013–2018	243 (27.6%)	442 (28.7%)	685 (28.3%)	
**Roadway alignment**				**<0.0001**
Straight	429 (48.8%)	899 (58.5%)	1328 (54.9%)	
Curve	449 (51.0%)	636 (41.4%)	1085 (44.9%)	
Missing	2	3	5	
**Roadway grade**				**0.0294**
Level	489 (55.7%)	924 (60.1%)	1413 (58.4%)	
Grade	389 (44.2%)	610 (39.7%)	999 (41.3%)	
Missing	2	4	6	
**Roadway surface type**				**0.0427**
Blacktop/bituminous/asphalt	808 (91.8%)	1443 (93.8%)	2251 (93.1%)	
Others**	71 (8.1%)	91 (5.9%)	162 (6.7%)	
Missing	1	4	5	
**Adverse weather**				**0.1466**
Yes	162 (18.4%)	321 (20.9%)	483 (20.0%)	
No	717 (81.5%)	1216 (79.1%)	1933 (79.9%)	
Missing	1	1	2	
**Vehicle characteristics**				
**Vehicle type**				**<0.0001**
Passenger cars	353 (40.1%)	958 (62.3%)	1311 (54.2%)	
Utility Vehicle	231 (26.3%)	191 (12.4%)	422 (17.5%)	
Pickups	262 (29.9%)	325 (21.1%)	587 (24.3%)	
Vans	30 (3.4%)	64 (4.2%)	94 (3.9%)	
Missing	4	0	4	
**Number in vehicle crash**				**<0.0001**
Single vehicle	770 (87.5%)	601 (39.1%)	1371 (56.7%)	
More than one vehicle	110 (12.5%)	937 (60.9%)	1047 (43.3%)	
**Airbag deployed**				**<0.0001**
Yes	343 (39.0%)	916 (59.6%)	1259 (52.1%)	
No	348 (39.6%)	244 (15.9%)	592 (24.5%)	
Unknown††	189	378	567	
**Vehicle age**				**0.8152**
≤ 10 years	483 (54.9%)	837 (54.4%)	1320 (54.6%)	
> 10 years	396 (45.0%)	700 (45.5%)	1096 (45.3%)	
Missing	1	1	2	

* Descriptive analysis used to compare rollover vs. nonrollover fatality counts and percentages. Missing values are not represented in the percentages.

† P-value for Chi-square test statistic was applied to binary variables. Cochran-Armitage trend test was applied to ordinal variables. Variables with p-value <0.20 in bold are selected for multivariable analysis.

§ Abbreviation: Driving While Impaired

¶ Includes “unknown,” “not reported,” and “no statutory speed limit,”

** Includes “Concrete,” “Brick/block,” “Slag, gravel, or stone,” “Dirt,” “Other” roadway surface type

†† Not available airbag deployed information.

**Table 3:** Adjusted Odds Ratios for Associations Between Risk Factors and Rollover Crashes*

Variables	Adjusted ORs and 95% CIs†	
	Model 1 (N= 2173)	Model 2 (N=1634)
**Driver characteristics**		
**Age**		
16–24	1.17 (0.92, 1.49)	1.37 (0.97, 1.91)
25–44	1	1
≥45	0.93 (0.76, 1.15)	0.80 (0.60, 1.07)
**Gender**		
Male	1.18 (0.96, 1.46)	0.98 (0.73, 1.30)
Female	1	1
**Blood Alcohol Concentration**		
0.00	1	1
0.01–0.07	1.32 (0.80, 2.18)	0.65 (0.32, 1.31)
≥0.08	**2.14 (1.74, 2.61)**	**1.56 (1.14, 2.14)**
**Restraint Use**		
Belted	1	1
Not belted	**1.70 (1.39, 2.08)**	1.22 (0.93, 1.61)
**Drug Test Result**		
Positive	1.20 (0.99, 1.45)	1.13 (0.87, 1.46)
Negative	1	1
**Environmental characteristics**		
**Speed Limit**		
30 or less		1
35 to 55		1.43 (0.91, 2.26)
60 or more		**4.06 (2.43, 6.78)**
**Land use**		
Rural		**1.39 (1.02, 1.89)**
Urban		1
**Day of week**		
Weekdays		1
Weekends		0.90 (0.69, 1.17)
**Time of day**		
Daytime		1
Nighttime		1.21 (0.91, 1.60)
**Roadway alignment**		
Straight		1
Curve		1.16 (0.90, 1.60)
**Roadway grade**		
Level		1
Grade		1.18 (0.92, 1.49)
**Pavement surface type**		
Blacktop/bituminous/asphalt		1
Others		1.18 (0.92, 1.52)
**Adverse weather**		
Yes		0.96 (0.71, 1.31)
No		1
**Vehicle characteristics**		
**Vehicle type**		
Passenger cars		1
Utility vehicle		**2.44 (1.76, 3.39)**
Pickups		**1.54 (1.12, 2.12)**
Vans		1.45 (0.79, 2.67)
**Number in vehicle crash**		
Single-vehicle crash		**8.37 (6.18, 11.34)**
More than one vehicle		1
**Airbag deployed**		
Yes		1
No		**2.71 (2.10, 3.51)**

* Significant ORs (95% CIs) are highlighted as they have a p-value <0.05 and OR does not include 1.

† Model 1 includes all driver characteristics (age group, gender, blood alcohol concentration, restraint use, and known drug test result). Model 2 includes all driver, environmental, and vehicle characteristics variables.

## References

[1] National Highway Traffic Safety Administration 2018 fatal motor vehicle crashes: overview National Center for Statistics and Analysis. No. DOT HS 812, 456 Accessed October 2019 Available at: https://crashstats.nhtsa.dot.gov/Api/Public/ViewPublication/812826

[2] Funk JR Cormier JM Manoogian SJ Comparison of risk factors for cervical spine, head, serious, and fatal injury in rollover crashes Accident Anal Prev 2012 45 67 74 10.1016/j.aap.2011.11.009 22269486

[3] Conroy C Hoyt DB Eastman AB Rollover crashes: predicting serious injury based on occupant, vehicle, and crash characteristics Accident Anal Prev 2006 38 5 835 42 10.1016/j.aap.2006.02.002 16540073

[4] Insurance Institute for Highway Safety (IIHS) Highway Loss Data Institute (HLDI). Fatality Facts 2019 Passenger vehicle occupants Available at: https://www.iihs.org/topics/fatality-statistics/detail/passenger-vehicle-occupants

[5] National Highway Traffic Safety Administration (NHTSA) Preview of motor vehicle traffic fatalities in 2019, 2020 National Highway Traffic Safety Administration Washington, DC No. DOT HS 813 021. Available at: https://crashstats.nhtsa.dot.gov/Api/Public/ViewPublication/813021

[6] National Highway Traffic Safety Administration (NHTSA) Characteristics of fatal rollover crashes 2002 National Highway Traffic Safety Administration Washington, DC Available at: https://crashstats.nhtsa.dot.gov/Api/Public/Publication/809438

[7] Farmer CM Lund AK Rollover risk of cars and light trucks after accounting for driver and environmental factors Accident Anal Prev 2002 34 2 163 73 10.1016/s0001-4575 01 00010 0 11829286

[8] Wen H Tang Z Zeng Y Zhang K A comprehensive analysis for the heterogeneous effects on driver injury severity in single-vehicle passenger car and SUV rollover crashes J Adv Transport 2020 1273605 10.1155/2020/1273605

[9] Wilson FA Stimpson JP Pagán JA Fatal crashes from drivers testing positive for drugs in the U.S., 1993–2010 Public Health Rep 2014 Jul–Aug 129 4 342 50 10.1177/003335491412900409 24982537 PMC4037460

[10] National Highway Traffic Safety Administration (NHTSA) Drug Involvement of Fatally Injured Drivers Traffic Safety Facts Accessed November 2010 United States Department of Transportation DOT HS 811 415 ed 2010 1 3 Available at: https://crashstats.nhtsa.dot.gov/Api/Public/ViewPublication/811415

[11] Brady JE Li G Trends in alcohol and other drugs detected in fatally injured drivers in the United States, 1999–2010 Am J Epidemiol 2014 179 6 692 9 10.1093/aje/kwt327 24477748 PMC3939850

[12] Azagba S Latham K Shan L Qeadan F Positive drug test trends in fatally-injured drivers in the United States from 2007 to 2017 Subst Abuse Treat Pr 2019 14 1 43 10.1186/s13011-019-0228-z PMC6815059 31653263

[13] NIDA Drugged Driving DrugFacts 2019 National Institute on Drug Abuse website. Available at: https://www.drugabuse.gov/publications/drugfacts/drugged-driving

[14] National Institute on Drug Abuse (NIDA) West Virginia Opioid Summary Revised March 2019. Available at: https://www.drugabuse.gov/opioid-summaries-by-state/west-virginia-opioid-summary

[15] National Highway Traffic Safety Administration Fatality Analysis Reporting System (FARS) Analytical User’s Manual 1975–2018 (Report No. DOT HS 812 827) Accessed September 2019 National Highway Traffic Safety Administration, U.S. Department of Transportation Washington, DC Available at: https://crashstats.nhtsa.dot.gov/Api/Public/ViewPublication/812827

[16] Hosseinpour M Yhaya AS Sadullah AF Ismail N Reza Ghadiri SM Evaluating the effects of road geometry, environment, and traffic volume on rollover crashes Transport 2016 31 2 221 232 10.3846/16484142.2016.1193046

[17] Khan IU Vachal K Factors affecting injury severity of single-vehicle rollover crashes in the United States Traffic Inj Prev 2020 21 1 66 71 10.1080/15389588.2019.1696962 31906717

[18] Dabbour E Using logistic regression to identify risk factors causing rollover collisions Int J Traffic Transport Engineering 2012 2 4 372 9 10.7708/ijtte.2012.2(4).07

[19] Zhu M Zhao S Gurka KK Kandati S Coben JH Appalachian versus non-Appalachian U.S. traffic fatalities, 2008–2010 Ann Epidemiol 2013 Jun 23 6 377 80 10.1016/j.annepidem.2013.04.001 23619016 PMC3689544

[20] National Highway Traffic Safety Administration (NHTSA) Rural/urban comparison of traffic fatalities Accessed April 2018 Report NO DOT HS 812 521 National Highway Traffic Safety Administration Washington, DC Available at: https://crashstats.nhtsa.dot.gov/Api/Public/ViewPublication/812521

[21] Lee J Abdel-Aty M Cai Q Wang L Analysis of fatal traffic crash-reporting and reporting-arrival time intervals of emergency medical services Transport Res Rec 2018 2672 61 71 10.1177/0361198118772724

[22] Ma L Zhang H Yan X Wang J Song Z Xiong H Smooth associations between the emergency medical services response time and the risk of death in road traffic crashes. J Transp Health 2019 12 379 391 10.1016/j.jth.2018.08.011

[23] Centers for Disease Control and Prevention Alcohol and other drug use among victims of motor vehicle crashes-West Virginia 2004–2005” MMWR-Morbid Mortal W 2006 55 48 1293 6 Available at: https://www.cdc.gov/mmwr/preview/mmwrhtml/mm5548a2.htm 17159830

