# Supplementary material for: Risk Factors Associated with Passenger Vehicle Fatal Rollover Crashes in West Virginia, 2001–2018
Source: J Appalach Health. 2021 Oct 25;3(4):45–59. doi: 10.13023/jah.0304.05 (PMC9183791; doi:10.13023/jah.0304.05)
Supplement: Supplementary file 1 [file 3.4.5-TANG-SUPP_TABLE.pdf]

Supplement Table 1. Sensitivity Analysis of Adjusted Odds Ratios for Associations Between Risk Factors and Rollover Crashes*

Variables	Adjusted ORs and 95% CIs†	
	Model 1<br>(N= 2,886)	Model 2<br>(N=2,119)
Driver characteristics		
Age		
16-24	1.19 (0.96, 1.46)	1.23 (0.91, 1.65)
25-44	1	1
≥45	0.95 (0.79, 1.14)	0.79 (0.61, 1.02)
Sex		
Male	1.17 (0.98, 1.40)	0.96 (0.75, 1.24)
Female	1	1
Blood Alcohol Concentration		
0.00	1	1
0.01-0.07	1.37 (0.92, 2.03)	1.07 (0.61, 1.90)
≥0.08	**2.05 (1.71, 2.45)**	**1.46 (1.10, 1.92)**
Restraint Use		
Belted	1	1
Not belted	**1.65 (1.39, 1.96)**	**1.29 (1.02, 1.64)**
Drug Test Result		
Positive	**1.24 (1.05, 1.46)**	1.12 (0.90, 1.41)
Negative	1	1
Environmental characteristics		
Speed Limit		
30 or less		1
35 to 55		**1.64 (1.10, 2.45)**
60 or more		**4.89 (3.11, 7.67)**
Land use		
Rural		**1.47 (1.12, 1.93)**
Urban		1
Day of week		
Weekdays		1
Weekends		0.94 (0.74, 1.19)
Time of day		
Daytime		1
Nighttime		1.16 (0.90, 1.49)
Roadway alignment		
Straight		1
Curve		1.24 (0.99, 1.55)
Roadway grade		
Level		1
Grade		1.12 (0.89, 1.40)
Pavement surface type		
Blacktop/bituminous/asphalt		1
Others		1.14 (0.73, 1.78)
Adverse weather		
Yes		0.82 (0.63, 1.08)
No		1

Continued in next page

Table 3 (Continued)

Variables	Adjusted ORs and 95% CIs†	
	Model 1 (N= 2,173)	Model 2 (N=1,634)
Vehicle characteristics		
Vehicle type		
Passenger cars		1
Utility vehicle		**2.20 (1.65, 2.92)**
Pickups		**1.83 (1.39, 2.42)**
Vans		1.60 (0.92, 2.77)
Number of vehicle crash		
Single-vehicle crash		**8.45 (6.48, 11.02)**
More than one vehicle		1
Airbag deployed		
Yes		1
No		**2.53 (2.03, 3.17)**

*The main purpose of sensitivity analysis is to estimate association between risk factors and fatal rollover crashes for all fatal drivers, including drivers died after 2 hours of fatal rollover crashes. Significant ORs (95% CIs) are highlighted as they have a p-value <0.05 and OR does not include 1.

†Model 1 includes all driver characteristics (age group, sex, blood alcohol concentration, restraint use, and known drug test result). Model 2 includes all driver, environmental, and vehicle characteristics variables.
